# Data supporting the comparative life cycle assessment of different municipal solid waste management scenarios

**DOI:** 10.1016/j.dib.2015.02.020

**Published:** 2015-03-20

**Authors:** Mohammad Ali Rajaeifar, Meisam Tabatabaei, Hossein Ghanavati

**Affiliations:** a Department of Biosystems Engineering, Faculty of Agriculture, University of Tabriz, Tabriz, Iran.; b Biofuel Research Team (BRTeam), Karaj, Iran; c Microbial Biotechnology and Biosafety Department, Agricultural Biotechnology Research Institute of Iran (ABRII), AREEO, 3135933151 Karaj, Iran; d Tim-Kian Co., Tehran, Iran

## Abstract

Environmental assessment of municipal solid waste (MSW) management scenarios would help to select eco-friendly scenarios. In this study, the inventory data in support of life cycle assessment of different MSW are presented. The scenarios were defined as: anaerobic digestion (AD, Sc-0), landfilling combined with composting (Sc-1), incineration (Sc-2), incineration combined with composting (Sc-3), and AD combined with incineration (Sc-4). The current article contains flowcharts of the different scenarios. Additionally, six supplementary files including inventory data on the different scenarios, data on the different damage assessment categories, normalization, and single scores are presented (Supplementary files 1–6). The analysis of the different scenarios revealed that the most eco-friendly scenario to be implemented in the future would be the combination of AD and incineration (Sc-4).

Specifications table

**Table:** 

Subject area	Waste management
More specific subject area	Life cycle assessment of municipal solid waste
Type of data	Excel tables, figures
How data was acquired	Using data imaging from a sufficient samples of inputs/emissions over an adequate period to even out normal fluctuations
Data format	Raw/analyzed
Experimental factors	Energy inputs/outputs and material/operation emissions
Experimental features	Inputs consumption and output production from different scenarios were measured during the year of study (Jan. 2013–Jan. 2014). Moreover, emissions to air/soil/water were collected based on the different operation or consumption of different materials in the scenarios.
Consent	N/a
Data source location	Tehran, Iran

**Table:** 

**Value of the data**
• The data open possibilities for municipalities to choose the most eco-friendly scenarios for municipal solid waste (MSW) treatment centers based on a specific region.
• The data could be of assistance in understanding how to organize the analyzed LCA data in order to choose the best MSW treatment scenario.
• Anaerobic digestion and incineration data presented herein could provide comprehensive information and could be used in future studies on MSW management scenarios.

## 1 Data, experimental design, materials and methods

**Fig. 1:**
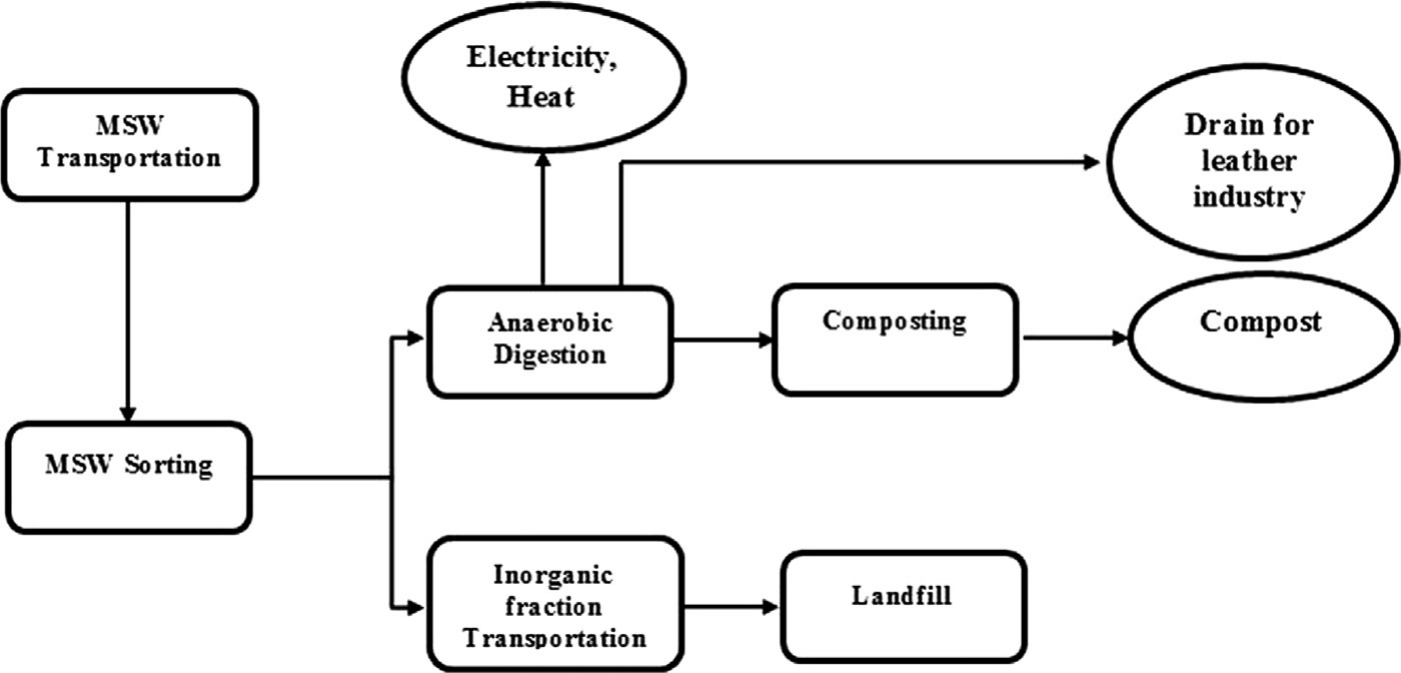
Flowchart of main steps involved in scnario Sc-0 (Currently in-use).

This paper presents data supporting the comparative life cycle assessment of different municipal solid waste management (MSW) scenarios. Tehran the capital of Iran and the largest metropolis in Western Asia with around 10 million inhabitants generating over 2,700,000 tons of MSW annually was selected as the case study. Currently, there are two current management scenarios in Tehran e.g. anaerobic digestion (AD) and landfilling, and the latter is the only option available to 21 out the 22 urban regions of Tehran. More specifically, 82% of the total MSW generated in Tehran is treated through landfilling. However, this scenario is fading gradually owing to the recent introduction of more advanced technologies such as AD. Moreover very recently, incineration scenarios have also been considered to be implemented by Tehran municipality. On such basis, five different scenarios were defined in this study, including the fading, currently in-use and future scenarios. The scenarios are presented in further details:

Scenario Sc-0 (Currently in-use scenario): In this scenario, MSWs are subjected to an AD process. As Fig. 1 shows, MSWs are transported to the treatment center, where the organic fraction are separated by a sorting process and are introduced to the AD plant. The inorganic fraction (rejected waste) is subjected to a landfilling process and recyclable materials such as aluminum, polyethylene terephthalate (PET), glass, iron sheet, cardboard, plastic bags and other plastics are separated for a recovery process. Finally, electricity and heat are generated through the AD process. There are 4 main steps involved in this scenario including: transportation, sorting, landfilling and AD. The inventory data for energy consumption and environmental emissions were collected for each stage (Supplementary file 1; Scenario Sc-0, Inventory Data.xlsx).

**Fig. 2:**
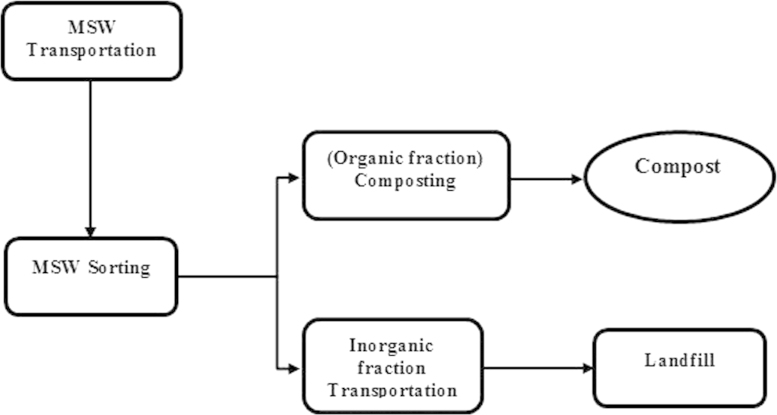
Flowchart of main steps involved in scenario Sc-1 (Fading scenario).

Scenario Sc-1 (Fading scenario): In this scenario, MSWs are subjected to a landfilling process in combination with a composting process (without biogas delivery from the landfill site). There are 4 main steps involved in this scenario including: transportation, sorting, landfilling and composting. The inventory data for energy consumption and environmental emissions were collected for each stage (Supplementary file 2; Scenario Sc-1, Inventory Data.xlsx) Fig 2.

**Fig. 3:**
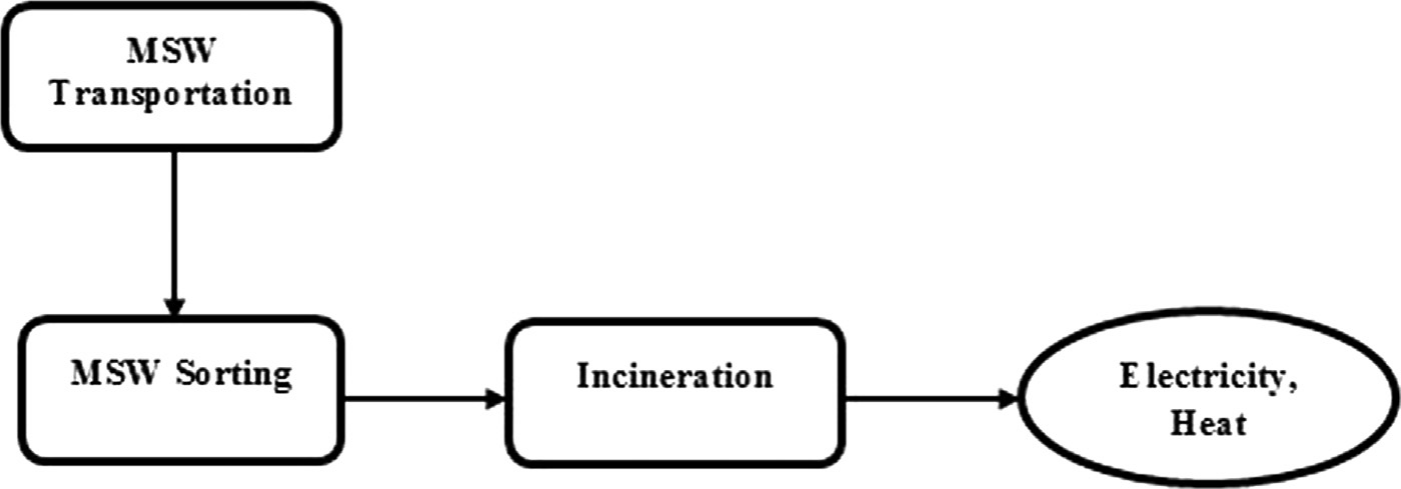
Flowchart of main steps encompasssed in scenario Sc-2 (Future scenario).

Scenario Sc-2 (Future scenario): This scenario represents incineration technique in which all the MSWs are incinerated after removing the recyclable materials (Fig. 3). Transportation, sorting and incineration are the major steps involved in this scenario. The inventory data for energy consumption and environmental emissions were collected for each stage (Supplementary file 3; Scenario Sc-2, Inventory Data.xlsx).

**Fig. 4:**
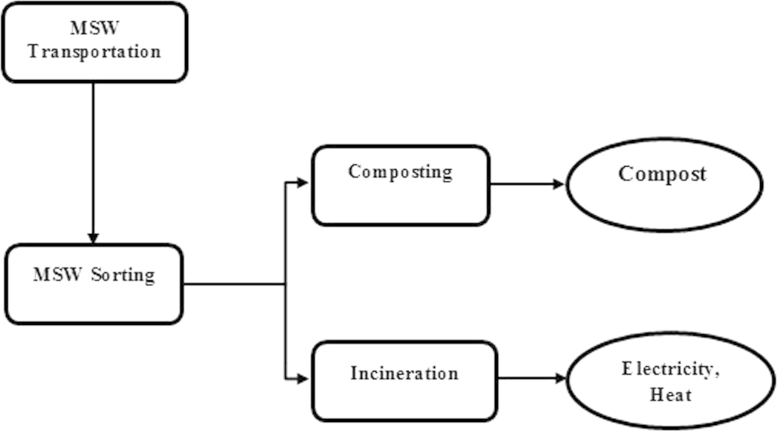
Flowchart of main steps involved in scenario Sc-3 (Future scenario).

Scenario Sc-3 (Future scenario): This scenario represents incineration in combination with composting process (Fig. 4). The only difference between this scenario and the scenario Sc-2 is that the organic fraction of MSW is subjected to an aerobic maturation in the composting process. There are 4 main steps involved in this scenario including: transportation, sorting, composting and incineration. The inventory data for energy consumption and environmental emissions were collected for each stage (Supplementary file 4; Scenario Sc-3, Inventory Data.xlsx).

**Fig. 5:**
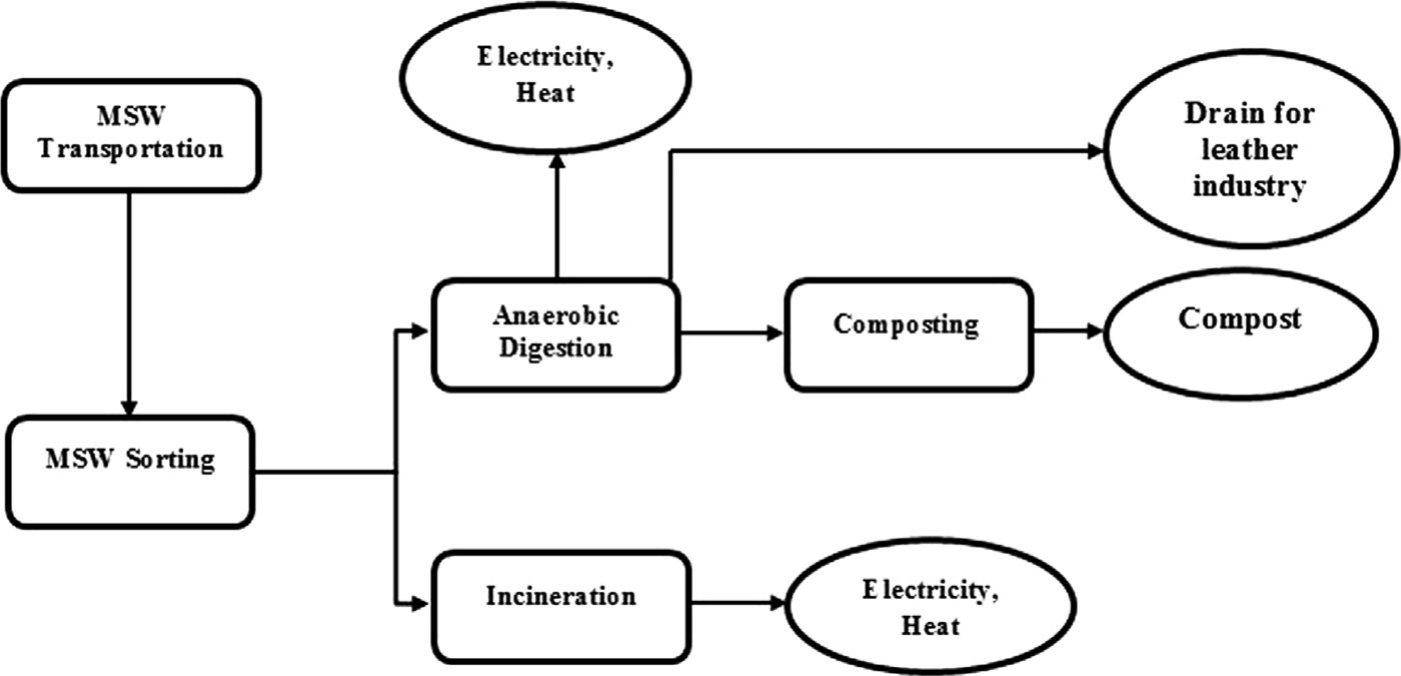
Diagram of the main steps included in scenario Sc-4 (Future scenario).

Scenario Sc-4 (Future scenario): This scenario is a combination of AD technique and incineration process (Fig. 5). MSWs are transported to the center and subjected to a sorting process. Organic matters are subjected to the AD process for biogas production and consequent production of electricity and heat. Rejected wastes are subjected to the incineration process for electricity production. Also, recyclable materials would be separated for a recovery process. Transportation, sorting, AD and incineration are the main steps involved in this scenario. The inventory data for energy consumption and environmental emissions were collected for each stage (Supplementary file 5; Scenario Sc-4, Inventory Data.xlsx)).

### 1.1 Functional unit and system boundaries

**Fig. 6:**
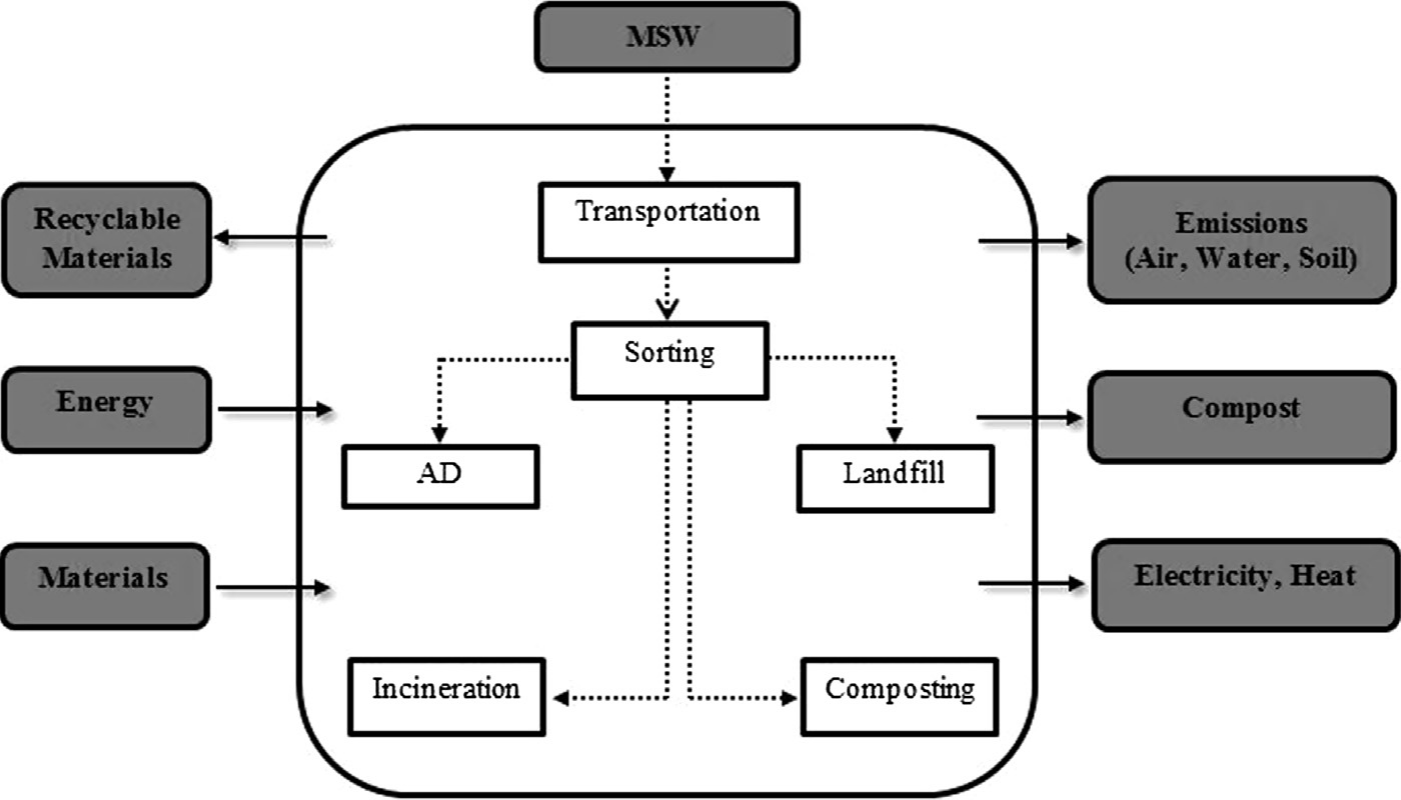
System boundries investigated in this study.

The functional unit of this study represents management of 1 tone of municipal solid waste in Tehran metropolise during the studying period (Jan. 2013–Jan. 2014). The function is the treatment of this amount of MSW, using different technologies in order to exploit energy, and/or to recycle wastes. Transportation, sorting, AD, landfilling, incineration and composting were considered as the scope of the present study. Fig. 6 shows the system boundaries of the study.

### 1.2 Waste composition and properties

The cornerstone of a solid waste LCA study is the knowledge on waste composition, chemical waste composition and other waste properties (calorific value, moisture content, etc.) [1,2]. Hence, waste composition and properties, chemical analysis of waste and analysis of rejected waste are presented in the Supplementary file 6 (Waste composition and properties.xlsx).

### 1.3 Energy aspects

Since the choice of electricity mix plays an important role in the waste LCA results [1], the electricity production mix for Iran was updated in this study and presented within the Supplementary file 6 (Iran electricity production mix.xlsx).

### 1.4 Life cycle impact assessment method

Among the different impact assessment methods, the Impact 2002+ method was used due to the fact that it is the mostly used models in Life cycle assessment of MSWs [3,4]. The Impact 2002+ method comprises four assessment methods: Ecoindicator 99 [5], CML [6], IMPACT 2002 [7] and IPCC [8]. The Impact 2002+ method includes 15 mid-points impact categories which are structured into four damage categories of Human Health, Ecosystem Quality, Climate Change and Resource Depletion [9]. SimaPro software version 8.1 was used to perform the LCA study along with its associated database (professional). In order to find the environmental impacts associated with the energy, transport and materials employed in the study, the Ecoinvent database v3.0 (2014) was used.

The overall assessment data are presented in (Supplementary file 6) which shows: (1) the contribution of each process from each scenario on different damage categories. (2) Each scenario contribution from each damage category. The analysis of the different scenarios revealed that the most eco-friendly scenario to be implemented in the future would be the combination of AD and incineration (Sc-4). Such analyses would assist in selecting the most appropriate procedures to convert biomass into value-added products such as various types of biofuels [10-12].
